# Automatic Epileptic Seizure Detection Using Scalp EEG and Advanced Artificial Intelligence Techniques

**DOI:** 10.1155/2015/986736

**Published:** 2015-01-29

**Authors:** Paul Fergus, David Hignett, Abir Hussain, Dhiya Al-Jumeily, Khaled Abdel-Aziz

**Affiliations:** ^1^Applied Computing Research Group, Liverpool John Moores University, Byrom Street, Liverpool L3 3AF, UK; ^2^The Walton Centre NHS Foundation Trust, Lower Lane, Fazakerley, Liverpool L9 7LJ, UK

## Abstract

The epilepsies are a heterogeneous group of neurological disorders and syndromes characterised by recurrent, involuntary, paroxysmal seizure activity, which is often associated with a clinicoelectrical correlate on the electroencephalogram. The diagnosis of epilepsy is usually made by a neurologist but can be difficult to be made in the early stages. Supporting paraclinical evidence obtained from magnetic resonance imaging and electroencephalography may enable clinicians to make a diagnosis of epilepsy and investigate treatment earlier. However, electroencephalogram capture and interpretation are time consuming and can be expensive due to the need for trained specialists to perform the interpretation. Automated detection of correlates of seizure activity may be a solution. In this paper, we present a supervised machine learning approach that classifies seizure and nonseizure records using an open dataset containing 342 records. Our results show an improvement on existing studies by as much as 10% in most cases with a sensitivity of 93%, specificity of 94%, and area under the curve of 98% with a 6% global error using a *k*-class nearest neighbour classifier. We propose that such an approach could have clinical applications in the investigation of patients with suspected seizure disorders.

## 1. Introduction

The epilepsies are a heterogeneous group of neurological disorders and syndromes characterised by recurrent, involuntary, paroxysmal seizure activity, which is typically associated with a clinicoelectrical correlate on the electroencephalogram (EEG). The diagnosis of epilepsy can be made, following two or more unprovoked seizures (http://www.who.int/). However, in the absence of a reliable witness account, diagnosis in the early stages of the disease can be challenging, which may delay initiation of treatment. Where there is clinical uncertainty, paraclinical evidence from the EEG can allow earlier diagnosis and treatment. However, EEG capture and interpretation are time consuming and costly because interpretation can currently only be performed by specialist clinicians, trained in EEG interpretation. This has led to a recent interest in automated seizure detection [1].

Although seizure semiology often gives clinical clues as to whether seizures are focal or generalised in onset and which lobe of the brain the seizure originated from, it is often more challenging to determine whether the seizure originated in the left or right hemisphere, in particular, in the case of temporal and occipital lobe epilepsies. In such cases or where there is clinical uncertainty, it is impossible to know, before performing the test, on which EEG channels seizure activity will be detected. This poses a problem when trying to generalise the detection of seizures across multiple subjects. Recent work on automated seizure detection from EEG recordings has focused on patient-specific predictors, where a classifier is trained and tested on the same person [2–5]. In this paper, the focus is on using EEG classification to generalise detection across all brain regions, in multiple subjects without a priori knowledge of the seizure focus.

The structure, of the remainder, of this paper is as follows.  Section 2 describes the principles of preprocessing EEG data.  Section 3 describes how features are extracted from EEG signals.  Section 4 discusses machine learning and its use in seizure and nonseizure classification, while Section 5 presents the approach taken in this paper for whole-brain seizure detection.  Section 6 describes the evaluation, Section 7 discusses the results, and conclusions are presented in Section 8.

## 2. Preprocessing of Electroencephalography Data

Electroencephalography is the term given to the technique of recording electrical activity resulting from ionic current flows generated by neurons in the brain [6]. Its main clinical application is in the evaluation of patients with suspected epilepsy.

Before analysis or classification occurs, EEG signals, in their raw form, need preprocessing. Preprocessing often includes filtering and artefact removal as recordings can contain unwanted noise mixed with the actual EEG energy/brain wave/signal. Artefacts can originate from various sources such as the subject, equipment, or the environment and consist of ocular artefacts, such as eye blinks; movement of the* EEG* sensors; and electromyogenic artefacts, caused by muscle movement. Artefacts are normally removed by eliminating certain frequencies from the EEG signal using high-pass, low-pass, band-pass, and notch filters [7].

One of the most common filters used in previous studies is a notch filter [8]. A notch filter removes any part of the signal that is at a specific frequency. Power line artefacts reside between 50 and 60 Hz and are removed when EEG frequencies above 60 Hz are used [9, 10]. However, there has been little justification for the use of higher frequencies [11, 12] because most brain activity occurs between 3 and 29 Hz. In support of this, Libenson [6] argues that EEG instruments rarely exceed 30–40 Hz and signals from cortically implanted electrodes rarely exceed 50 Hz due to electrical noise and other artefacts such as muscle movement. For these reasons, Blanco et al. [13] use an upper cut-off frequency of 40 Hz and [14] use an upper range of 35 Hz, whereas Greene et al. [15] filter out frequencies above 34 Hz stating that the frequency range 2–20 Hz provides the best discrimination between seizure and nonseizureevents. In other studies, Wang et al. [16] use a frequency range between 8 and 32 Hz and, in [17], frequencies above 30 Hz are filtered, whereas Yuan et al. [18] split signals into different frequency bands using bandpass filters for theta (*θ*: 4 ≤ *f* ≤ 8 Hz), alpha (*α*: 8 ≤ *f* ≤ 12 Hz), and beta (*β*: 12 ≤ *f* ≤ 25 Hz) to ensure that only specific physiological data is considered.

At the lower end of the frequency spectrum, the most common cut-off filter value is around 0.5 Hz [19–22]. In [6], the author argues that there is no cerebral activity below 0.5 Hz and what little there is cannot be reliably observed in conventional EEG recordings. In fact, the majority of signals below 0.5 Hz usually represent motion or other electrical activity.

## 3. Feature Extraction from Electroencephalography Signals

The collection of raw EEG signals is always temporal. However, for analysis and feature extraction purposes, translation, into other domains, is possible and often required. These include frequency representations, via Fourier transform, [19–22] and wavelet transform [22–27]. The advantage of frequency-related parameters is that they are less susceptible to signal quality variations, due to electrode placement or the physical characteristics of subjects [28]. In order to calculate these parameters, a transform from the time domain is required, that is, using a Fourier transform of the signal. In several of the studies reviewed, power spectral density (PSD) is used, in order to obtain frequency parameters.* Peak Frequency* is one of the features considered in many studies. It describes the frequency of the highest peak in the PSD. During a seizure, EEG signals tend to contain a major cyclic component which shows itself as a dominant peak in the frequency domain [29].* Peak Frequency* has been used along with other features to achieve high classification accuracy. In one example, Aarabi et al. used* Peak Frequency* along with sample entropyand other amplitude features to detect epileptic seizures and achieved a sensitivityof 98.7% and a false detection rateof 0.27 per hour [30].

While Tzallas et al. found that* Peak Frequency*, along with 15 other features, provided accuracies between 78.11% and 86.18% when classifying transient events in EEG recordings [31], in [15], it was found that* Peak Frequency* only achieved an accuracy of 54.06%. A possible explanation for low accuracies could be that the frequency of peaks tends to decay over time. If the window that the* Peak Frequency* is extracted from is too large, this decaying of the peak could explain why some authors have experienced less accuracy when using only the* Peak Frequency *to detect seizures [32].

Wang and Lyu [33] found that median frequency displayed significant differences between seizure and nonseizure patients. By segmenting the EEG signal into five separate frequency bands for delta (*δ*: 0.5 ≤ *f* ≤ 4 Hz), theta (*θ*: 4 ≤ *f* ≤ 8 Hz), alpha (*α*: 8 ≤ *f* ≤ 12 Hz), beta (*β*: 12 ≤ *f* ≤ 25 Hz), and gamma (*γ*: 25 ≤ *f*), it was possible to predict 79 of 83 seizures with a sensitivity value of 95.2%. In other works, Päivinen et al. [34] used linear and nonlinear features for detecting seizures and found that a combination of the two achieved the best results.

Root mean square (RMS) has been considered a useful feature for distinguishing between seizures and nonseizure events. RMS measures the magnitude of the varying quantity and is a good signal strength estimator in EEG frequency bands [35, 36]. In a study on neonatal seizure detection [15], 21 features for seizure classification were compared, which saw RMS achieve an overall accuracy of 77.71%, outperforming the other features studied. However, the figure was reportedly lower than that in another study [37] where* RMS* was used in conjunction with other features, rather than as a single feature.

Entropy has been used as a measure of the complexity or uncertainty, of an EEG signal, where the more chaotic the signal is, the higher the entropy is [15, 29]. There are two kinds of entropy estimators: spectral entropies, which use the amplitude of the power spectrum, and signal entropies, which use the time series directly [38]. Many authors agree that, during a seizure, the brain activity is more predictable than during a normal, nonseizure phase and this is reflected by a sudden drop in the entropy value [15, 30, 39–41]. In [38], four entropy measures were used, Shannon spectral entropy, Renyi's entropy, Kolmogorov-Sinai entropy, and approximate entropy, and over 90% classification accuracy was achieved. Wavelet entropy, sample entropy, and spectral entropy were compared in [42] in which accuracies between 79% and 99.8% were reported. In another similar study, using only approximate entropy, accuracies of 100% were achieved [43]. Several other studies produced comparatively high overall accuracies [44–46]. While [47] found that entropy features gave much lower classification accuracies between 54.5% and 76.3%, it was not clear why accuracies were low. However, one possible reason is the lack of data preprocessing. If EEG artefacts are not removed from seizure phases, this could make the seizure signal appear to be more complex and give the EEG signal a look more akin to anonseizure phase.

Energy is a measure of the EEG signal strength. Rather than looking at the energy of the whole EEG signal, the energy distribution across frequency bands has been used in seizure detection [48]. The study found that delta and theta frequency bands saw a much larger distribution of energy during a seizure compared to normal EEG, whereas the alpha, beta, and gamma frequency bands saw a lower energy distribution during a seizure. Using the energy distribution per frequency band as a feature achieved an overall accuracy of 94%. In [47], the results show that using energy as a feature produced classification accuracies between 92% and 99.81%. In a similar study, energy was also used along with entropy and standard deviation [49]. They were evaluated in isolation and combined together, with the best feature being energy with an overall accuracy of 91.2%.

Correlation dimension has been investigated as a correlation measure in several studies, which is a nonlinear univariate, widely used to measure fractal dimension. Fractal dimension measures the complexity of the EEG signal, in other words, the regularity and divergence of the signal [50, 51]. In [52], correlation dimension and five other features for seizure prediction of focal neocortical epilepsy produced reasonably good results with 90.2% for sensitivity and 97% for specificity. However, when looking specifically at the correlation dimension, they found conflicting results, where correlation dimension dropped in 44.9% of seizures and increased in the preictal phase in 44.9% of seizures. They also found that there were stronger dimension changes in the remote channels compared with those near seizure onset. It should be noted that the data preprocessing was minimal as the method for calculating the correlation dimensions tolerates a certain amount of noise. In addition, as their study is concerned with identifying the preictal state with the intention of predicting seizures, it differs from the current work, which is only interested in detecting the seizure retrospectively by classifying blocks of data as seizure or nonseizure.

In [53], correlation dimension and the largest Lyapunov exponent were studied to determine their ability to detect seizures. The study showed that neither measure on its own was useful for the task but did work better, when they were used together. They also noted that correlation dimension was only useful when applied to the frequency subbands (delta, theta, alpha, beta, and gamma), and not on the entire 0–60 Hz frequency spectrum that was used in the study. The authors concluded that changes in dynamics are not spread out across the entire spectrum but are limited to certain frequency bands. In a comparative study, [38] explored the use of correlation dimension, along with Hurst exponent, largest Lyapunov exponent, and entropy, to distinguish seizures from normal EEG. The results report an overall accuracy of 90% [54]. Meanwhile, [55] questions the use of correlation dimension and argues that it only reflects the change in variance and that there was little justification for its use over the simpler linear measure of variance.

Skewness is a third-order statistical moment, and kurtosis is the fourth [34]. Along with the first- and second-order moments, mean and variance, respectively, the four statistical moments provide information on the amplitude distribution of a time series. Specifically, skewness and kurtosis give an indication of the shape of the distribution [56]. Khan et al. use skewness and kurtosis, along with normalised coefficient of variation, for seizure detection in paediatric patients. They managed to detect all 55 seizures from a subset of 10 patients, achieving 100% sensitivity with a false detection rate of 1.1 per hour. Päivinen et al. examined spectral skewness and spectral kurtosis and found a high correlation between skewness and kurtosis. In their study, they rejected kurtosis arguing that it is of a higher order and thus more sensitive to noise. They concluded that a combination of linear and nonlinear features was best suited to seizure detection.

## 4. Seizure Detection and Classification

The first results in seizure detection and classification date back to 1979 [57]. Gotman et al. investigated the automatic recognition of interictal epileptic activity in prolonged EEG recordings using a spike and sharp wave recognition method [57–59]. This work lead to the investigation of functional magnetic resonance imaging (fMRI) and the correlation between cerebral hemodynamic changes and epileptic seizure events visible in EEG [60]. In 2013, stereoelectroencephalography (sEEG) using high frequency activities in the wavelet domain was proposed [61]. While the detection sensitivity was high (86%) and the specificity was acceptable (0.47/h), the detection delay is long (mean delay 16.2 s).

Since 1979, computer algorithms and visualisation techniques have played a central role in the analysis of EEG datasets. However, today, there is significant interest in classification algorithms. The most common classifier used to distinguish between seizure and nonseizure events has been the support vector machine (SVM). Using the Children's Hospital Boston-Massachusetts Institute of Technology (CHB-MIT) database and a patient-specific prediction methodology, the study in [62] used a SVM classifier on EEG recordings from 24 subjects. The results show that a classification accuracy of 96% and 96% for sensitivity were produced, with a false-positive rate of 0.08 per hour. While the results are encouraging, the approach is personalised to the individual. In other words the approach cannot be generalised across more than one patient. In a similar study, five patient records from the CHB-MIT dataset containing a total of 65 seizures were evaluated using a linear discriminant analysis classifier [63]. The results show that 83.6% was achieved for sensitivity, 100% for specificity, with an overall accuracy of 91.8%. There are two main issues with this study. The first is that the classifier is much more sensitive to nonseizures than seizures; failing to detect a seizure is more problematic than failing to detect a nonseizure. Second, the focus of the study is personalised to the individual and is incapable of being generalised across a wider population. Nasehi and Pourghassem [64] used the same CHB-MIT dataset with a particle swarm optimisation neural network (*PSONN*) which produced 98% for sensitivity and a false-positive rate of 0.125 per hour. This approach is much more sensitive to seizures than many of the studies reviewed in this paper. Yet, again, the approach is person specific rather than generalised across a wider population.

In [43], 100 seizure segments and 100 nonseizure segments were used to train a SVM classifier. The results show that 100% was obtained for sensitivity, specificity, and overall accuracy. Meanwhile, Nicolaou and Georgiou [65] carried out a similar study using the BONN dataset [43] and an SVM classifier, with 94.38% for sensitivity, 93.23% for specificity, and an overall accuracy of 86.1%. In a similar study, Übeyli [66], who also used the BONN dataset [43] and an SVM classifier, produced 99.25% for sensitivity, 100% for specificity, and 99.3% for overall accuracy. Extending this study, Übeyli evaluated seven different classifiers with the SVM proving the best-performing classifier with similar results produced to those in the original study [67]. The worst performing classifier was the multilayer perceptron neural network, which achieved 90.48% for sensitivity, 97.45% for specificity, and 90.48% for overall accuracy.

Acharya et al. focused on using entropies for EEG seizure detection and seven different classifiers [68]. The best-performing classifier was the Fuzzy Sugeno Classifier, which achieved 99.4% for sensitivity, 100% for specificity, and 98.1% for overall accuracy. The worst performing classifier was the Naïve Bayes Classifier, which achieved 94.4% for sensitivity, 97.8% for specificity, and 88.1% for accuracy. In [69], the decision tree classifier was used and achieved an average sensitivity of 99.24%, specificity of 98.76%, and accuracy of 99.02%.

The FRE (https://epilepsy.uni-freiburg.de/) dataset has featured in several studies, which contains EEG data from a number of patients, similar to the CHB-MIT database. However, it only has six channels, three close to the focus of the seizure and three further away. Using the* FRE* dataset, Yuan et al. presented a patient-specific seizure detection system and an extreme machine-learning algorithm to train a neural network [70]. Twenty-one seizure records were used to train the classifier and 65 for testing. The results show that the system achieved an average of 91.92% for sensitivity, 94.89% for specificity, and 94.9% for overall accuracy. Using the same dataset, Williamson et al. [71] used a SVM to classify EEG recordings from 18 of the 21 patients in the dataset. The results show an average sensitivity of 90.8% and a false-positive rate of 0.094 per hour. Park et al. [72] adopted a similar configuration and classifier and achieved 97.5% for sensitivity and a false-positive rate of 0.27 per hour. While Patnaik and Manyam [73] used a feed-forward back propagation artificial neural network on the 21 subjects from the* FRE* dataset, classification was performed on a patient-specific basis and the results, per patient, ranged from 98.32 to 99.82% for specificity and between 87.73 and 93.8% for sensitivity.

Patel et al. [74] proposed a low power, real-time classification algorithm, for detecting seizures in ambulatory EEG. The study compared linear discriminant analysis (LDA), quadratic discriminant analysis (QDA), Mahalanobis discriminant analysis (MDA), and SVM classifiers on 13 subjects from the FRE dataset. The results show that the LDA gave the best results when trained and tested on a single patient, with 94.2% for sensitivity, 77.9% for specificity, and 87.7% for overall accuracy. When generalised across all subjects, the results show 90.9% for sensitivity, 59.5% for specificity, and 76.5% for overall accuracy.

In a similar study, Acir and Güzeliş used SVM classifier to detect epileptic spikes [75]. The dataset used to evaluate their methodology was from the Neurology Department of Dokuz Eylul University Hospital, Izmir, Turkey, and consisted of 25 patients with one EEG record each, 18 used for training and 7 for testing. Their approach achieved 90.3% for sensitivity, 88.1% for specificity, and a 9.5% false detection rate. While an SVM classifier was considered to discriminate between preictal and nonpreictal states in [76], the authors used a 22 linear univariate feature space extracted from six EEG recordings for each of the 10 patients from the European database on epilepsy. Their approach could detect 34 of the 46 seizures achieving a sensitivity of 73.9% and a false prediction rate of 0.15/hour.

## 5. Generalisation of Epileptic Seizure Detection

Despite the advances within the last twenty years in the EEGseizure detection and prediction field, generalised detection approaches remain relatively poor. This is especially true when compared to patient-specific studies as discussed. Given this poor success, it may be easier to utilise an empirical backward looking, “data mining” or “brute force” approach. This is opposed to a forward-looking, conceptual model approach, in order to find features that best describe epilepsy.

The aim of most studies in EEG detection has been to detect patient-specific focal seizures, rather than predicting general seizures across a much bigger population. As Shoeb [5] explains, a seizure EEG pattern is specific to a particular patient. The main reason for this is that focal seizures can occur in any part of the brain, and, therefore, can only be detected in the EEG on specific channels. A classifier trained on a patient who experiences focal seizures in the occipital lobes, for example, would be trained on features from channels, including electrodes* O*,* O1*, and* O2*, as these would be the channels from the area of the seizure and, therefore, best at detecting the seizure. However, these trained classifiers achieve low sensitivity if they are tested on a patient who experiences focal seizures in the frontal lobes, as the channels around the focus of the seizure have not been used to train the classifiers.

In order to improve on earlier studies, using the CHB-MIT dataset, we aimed to discriminate between seizure and nonseizure EEGs across a group of 22 subjects with seizures occurring in different brain regions.

### 5.1. Methodology

The CHB-MIT dataset is a publicly available database from physionet.org that contains 686 scalp EEG recordings from 22 patients treated at the Children's Hospital in Boston. The subjects had anticonvulsant medications withdrawn and EEG recordings were taken for up to several days after.

Twenty-three sets of EEG recordings from 22 patients (5 males, 17 females), aged between 1.5 and 22 years (mean, SD), are contained within the dataset (one patient has two sets of EEG recordings 1.5 years apart).

Most of the recordings are one hour long, although those belonging to case 10 are two hours long and those belonging to cases 4, 6, 7, 9, and 23 are four hours long. Records that contain at least one seizure are classed as seizure records and those that contain no seizures as nonseizure records. Of the 686 records, 198 records contain seizures.

Although the description supplied with the dataset states that recordings were captured using the international 10–20 system of EEG electrode positions and nomenclature, it was found that 17 of the files that contained seizures had different channel montages to the rest of the seizure files. Therefore, these 17 records have been excluded from this study, leaving 181 seizure files. A further 10 records were removed from the dataset due to a large number of not a number (NaN) elements. The remaining 171 seizure records contain the length of the recording (in seconds) in the first column, followed by the 23 EEG channels in columns 2–24.  Table 1 shows the subject information as well as the number of seizures used in the study.

The final dataset used in this study was constructed from 60-second data blocks, comprising the ictal period (seizure), which were extracted from 171 seizure files, and 171 data blocks were randomly extracted from nonseizure files. The classifiers were then trained on all patient records and, therefore, classification is generalised across all subjects using features from channels that capture the EEG in all parts of the brain.

#### 5.1.1. Data Preprocessing

In the CHB-MIT database, each record was sampled at 256 Hz with 16-bit resolution. Signals were recorded simultaneously through twenty-three different channels (*FP1-F7, F7-T7, T7-P7, P7-O1, FP1-F3, F3-C3, C3-P3, P3-O1, FZ-CZ, CZ-PZ, FP2-F4, F4-C4, C4-P4, P4-O2, FP2-F8, F8-T8, T8-P8, P8-O2, P7-T7, T7FT9, FT9-FT10, FT10-T8, *and* T8-P8*), via 19 electrodes and a ground attached to the surface of the scalp. A number of records contained dashes (missing data) in the original data; no explanation is given to why the data contains dashes. However, possible reasons could be that there were errors in the recording phase or the occurrence of physiological symptoms, such as sweat interference with the electrodes or body movement. Each zero was found and replaced with a 256-point window (50% on either side of the zero) and was replaced with the mean value. Other data removed from the segments include electrocardiograph (*ECG*) signals and vagal nerve stimulus (*VNS*).

A bandpass filter was applied to each of the 543 EEG segments to extract the EEG data in each of the frequency bands. Second order Butterworth filters were used as they offer good transition band characteristics at low coefficient orders; thus, they can be implemented efficiently [2]. This results in four columns of additional data: delta (*δ*: 0.5 ≤ *f* ≤ 4 Hz), theta (*θ*: 4 ≤ *f* ≤ 8 Hz), alpha (*α*: 8 ≤ *f* ≤ 12 Hz), and beta (*β*: 12 ≤ *f* ≤ 25 Hz). In other words, each segment contains 115 columns of data for each of the original channel data.

#### 5.1.2. Feature Definition

Several features based on our findings in the literature are utilised in this study and are formally described. Each feature is ranked based on its discriminative capabilities using feature-ranking algorithms and principle component analysis.

The frequency domain features were extracted from the time-series signal using PSD. In this study, PSD is defined as the Fourier transform of the autocorrelation sequence of the times series. The Fourier transform *X*(*f*) of the signal *x*(*t*) is defined as

(1) $$\begin{array}{c} X\left(f\right)=\overset{+\infty }{\underset{-\infty }{\int }}x\left(t\right)e^{-j2\pi ft}dt\;-\infty <f<+\infty , \end{array}$$

where *X*(*f*) contains the information for the signal and *x*(*t*) is obtained from *X*(*f*) using the inverse of the Fourier transformation:

(2) $$\begin{array}{c} x\left(t\right)=\overset{+\infty }{\underset{-\infty }{\int }}X\left(f\right)e^{j2\pi ft}dt\;-\infty <f<+\infty . \end{array}$$

*Peak Frequency *is one of the features considered in many studies to have good discriminative capabilities and describes the frequency of the highest peak in the PSD*. Peak Frequency *is formally described as

(3) $$\begin{array}{c} f_{\max}=\arg\left(\frac{f_{s}}{N}\overset{N-1}{\underset{i=0}{\max}}P\left(i\right)\right), \end{array}$$

where *f*_*s*_ and *N* describe the sample frequency and the number of samples, respectively. Conversely, median frequency is used to estimate the typical frequency present in the signal and is regarded in the literature as a useful feature in EEG research. Median frequency is defined as

(4) $$\begin{array}{c} f_{\text{med}}=i_{m}\frac{f_{s}}{N},\;\;\;\overset{i=i_{m}}{\underset{i=0}{\sum }}P\left(i\right)\dot{=}\overset{i=N-1}{\underset{i=i_{m}}{\sum }}P\left(i\right). \end{array}$$

The median frequency is defined as the midpoint in the frequency power spectrum where the sum of the points on each side is equal. RMS is also used in this study as a signal strength estimator in EEG frequency bands. It provides a measure of the magnitude of the varying quantity and is defined as

(5) $$\begin{array}{c} \text{RMS}=\sqrt{\frac{1}{N}\overset{N-1}{\underset{i=0}{\sum }}x{\left(i\right)}^{2}}, \end{array}$$

where a signal represented by a time-series *x*(*t*) can be calculated as the root of the mean of the squares for all samples in the signal. Measuring the complexity of the signal is regarded as an important feature, which can be calculated using sample entropy. In other words, sample entropy calculates the uncertainty of an EEG signal. It is described as

(6) $$\begin{array}{c} \text{sampEmp}=\overset{N}{\underset{i=1}{\sum }}\left(X_{i}*\log\left(X_{i}^{2}\right)\right), \end{array}$$

where *N* is the length of the time series and *X*_*i*_ is the *i*th sample of the EEG signal. Signal energy is also an important feature and is useful for measuring the EEG signal strength in different frequency bands. It is defined as the sum of the squared magnitude of the samples:

(7) $$\begin{array}{c} E=\overset{N}{\underset{k=1}{\sum }}x_{k}^{2}. \end{array}$$

The correlation dimension feature is a useful measure of the regularity and divergence of a signal, that is, its complexity. It is proportional to the probability of the distance between two points on a trajectory being less than some *r*:

(8) $$\begin{array}{c} C_{\dim}=\underset{r\to \infty }{\lim}\frac{\log\left(C\left(r\right)\right)}{\log\left(r\right)}, \end{array}$$

where

(9) $$\begin{array}{c} C\left(r\right)=\underset{M\to \infty }{\lim}\frac{1}{M^{2}}\overset{M}{\underset{i=1}{\sum }}\;\overset{M}{\underset{j=i+1}{\sum }}\theta \left(r-\left|y\left(i\right)-y\left(j\right)\right|\right),\\ \theta \left(r-1\left|y\left(i\right)-y\left(j\right)\right|\right)=\left\{\begin{array}{cc} 1\text{:} & \left(r-\left|y\left(i\right)-y\left(j\right)\right|\right)\geq 0\\ 0\text{:} & \left(r-\left|y\left(i\right)-y\left(j\right)\right|\right)\leq 0. \end{array}\right. \end{array}$$

Skewness and kurtosis are useful for providing information on the amplitude distribution of a time series. In other words, they indicate the shape of the distribution. Skewness is defined as

(10) $$\begin{array}{c} s=\frac{{E\left(X-\mu \right)}^{3}}{\sigma^{3}}, \end{array}$$

where *E*(*x*) is the expected value of some variable *x*, *μ* is the mean, and *σ* is the standard deviation of the signal. Kurtosis is defined as

(11) $$\begin{array}{c} k=\frac{{E\left(x-\mu \right)}^{4}}{\sigma^{4}}. \end{array}$$

#### 5.1.3. Feature Selection

The literature reports that median frequency, sample entropy, and root mean square have the most potential to discriminate between seizure and nonseizure records. To validate these findings, the discriminant capabilities of each feature are determined using several measures: statistical significance, principal component analysis(PCA) [77], linear discriminant analysis independent search (LDAi) [77], linear discriminant analysis forward search(LDAf) [77], linear discriminant analysis backward search(LDAb) [77], and gram-Schmidt (GS) analysis [78]. Using these measures, the top 20 uncorrelated features were extracted from all regions of the EEG scalp readings (region-by-region feature extraction is considered later in the paper).

The uncorrelated feature sets were used with several classification algorithms to determine which set of features produced the highest area under the curve (AUC).  Table 2 shows that the best results obtained were from the linear discriminant analysis backward search technique with an AUC of 91%. This was followed closely by statistical *p* and *q*-*values* with AUC values of 90% and 89%, respectively.

Using PCA, we extracted the top five uncorrelated features from each of the five regions covered by the EEG scalp electrodes using the linear discriminant backward search technique (because it produced the highest AUC value of 91%). This ensures that each region is represented without the bias from all other regions and allows classifiers to detect focal seizures in different parts of the brain. The channels are grouped by region as shown in Table 3.

The top five features per region were selected based on their rank determined by the linear discriminant backward search technique, creating five feature sets containing five features each. These are combined to produce a set of 25 features as shown in Table 4.

**Figure 1:**
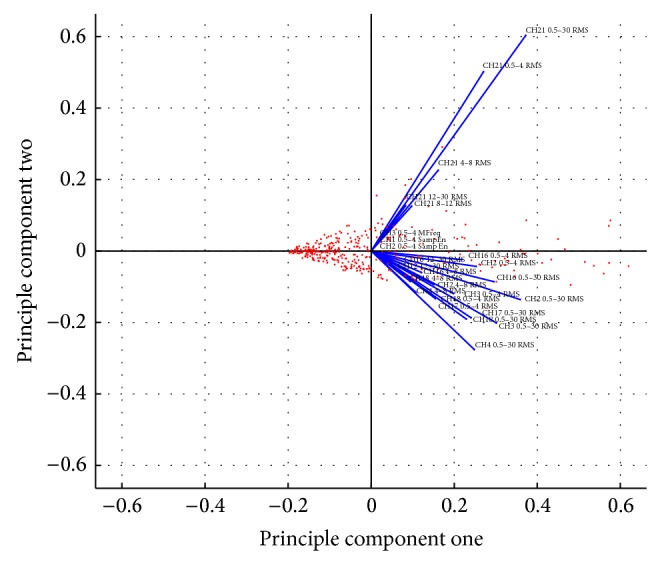
PCA for RMS feature discrimination.

Figure 1 shows that several RMS and median frequency features, from different channels and frequency bands, appear along the principal component. This is consistent with the findings in [33–36].

In summary, PCA makes a very strong case for the use of root mean square on different channels and frequency bands.

The features extracted using the generalised and region-by-region approach will be used to evaluate the capabilities of several classifiers considered in this study.

#### 5.1.4. Synthetic Minority Oversampling

The number of observations in this study is relatively low, and it would be useful to compare an oversampled dataset with the original dataset. In order to address this issue, it is necessary to resample the CHB-MIT dataset. In this study, the classes are balanced. However, resampling is used to generate additional observations for both seizure and nonseizure records.

Several studies have shown that the synthetic minority oversampling technique (SMOTE) has effectively solved the class skew problem [79–84]. In this study, SMOTE has been utilised to oversample both the seizure and nonseizure classes in order to generate new synthetic records (observations) along line segments joining the* k*-classnearest neighbours. This forces the decision tree region of the minority class to become more general and ensures that the classifier creates larger and less specific decision regions, rather than smaller specific regions. In [85], the authors indicated that this approach is an accepted technique for solving the problem related to unbalanced datasets and, in this study, the validity of this technique to increase the number of observations for both seizure and nonseizure classes is evaluated.

#### 5.1.5. Classification

Following an analysis of the literature, the study in this paper adopts simple yet powerful algorithms, as shown in Table 5.

These include the linear discriminant classifier (LDC), quadratic discriminant classifier (QDC), uncorrelated normal density-based classifier (UDC), polynomial classifier (POLYC), logistic classifier (LOGLC),* k*-class nearest neighbour (KNNC), decision tree (TREEC), Parzen classifier (PARZENC), and the support vector machine (SVC) [86]. The linear, quadratic, and uncorrelated normal density-based classifiers are all density-based classifiers. The LDC is particularly useful when two classes are not normally distributed and where monotonic transformations, of posterior probabilities, help to generate discriminant functions. The QDC assumes that the classes are normally distributed with class specific covariance matrices, thus allowing a set of optimal discriminant functions to be obtained. The UDC works in a similar way to the QDC classifier using computation of a quadratic classifier between the classes by assuming normal densities with uncorrelated features. The QDC takes decisions by assuming different normal distributions of data that lead to quadratic decision boundaries.

## 6. Evaluation

This section presents the classification results for seizure and nonseizure records using the CHB-MIT database. A feature set is extracted from the raw signal frequency bands; delta (*δ*: 0.5 ≤ *f* ≤ 4 Hz), theta (*θ*: 4 ≤ *f* ≤ 8 Hz), alpha (*α*: 8 ≤ *f* ≤ 12 Hz), and beta (*β*: 12 ≤ *f* ≤ 25 Hz) are used with an 80% holdout technique and* k-*fold cross-validation. The initial evaluation provides a base line for comparison against all subsequent evaluations, considered in this section.

### 6.1. Results Using Top Twenty Uncorrelated Features Ranked Using LDA Backward Search Feature Selection

In this evaluation, the top twenty uncorrelated features are extracted from each of the frequency bands within each of the EEG channels and used to train and test nine classifiers. The performance for each classifier is evaluated using the sensitivity, specificity, mean error, standard deviation, and AUC values with 100 simulations that use randomly selected training and testing sets.

#### 6.1.1. Classifier Performance

The first evaluation uses all the seizure and nonseizure blocks from all subjects in the CHB-MIT dataset (171 seizures and 171 nonseizures).  Table 6 shows the mean averages obtained over 100 simulations for the sensitivity, specificity, and AUC.

As shown in Table 6, the sensitivities (seizure), in this initial test, are lower for all classifiers. This is interesting given that the number of seizureand nonseizure blocks is equal. One possible reason for this is that the ictal length across the 171 records was 60 seconds. However, in the CHB-MIT records ictal periods ranged between 6 and 752 seconds. It is possible that some ictal blocks resemble nonseizure records resulting in misclassification (particularly blocks that contain shorter runs of ictal data).  Table 7 compares the holdout results with the* k*-fold cross-validation technique using 5-fold and one and 100 iterations, respectively. The results show that all techniques are able to achieve a classification error, lower than the base-rate error of 50% (i.e., 171/342).

Despite a reasonably low error rate using the holdout technique, the* k*-fold cross-validation results slightly improved the error rates for some classifiers. However, these results were not statistically significant.

#### 6.1.2. Model Selection

**Figure 2:**
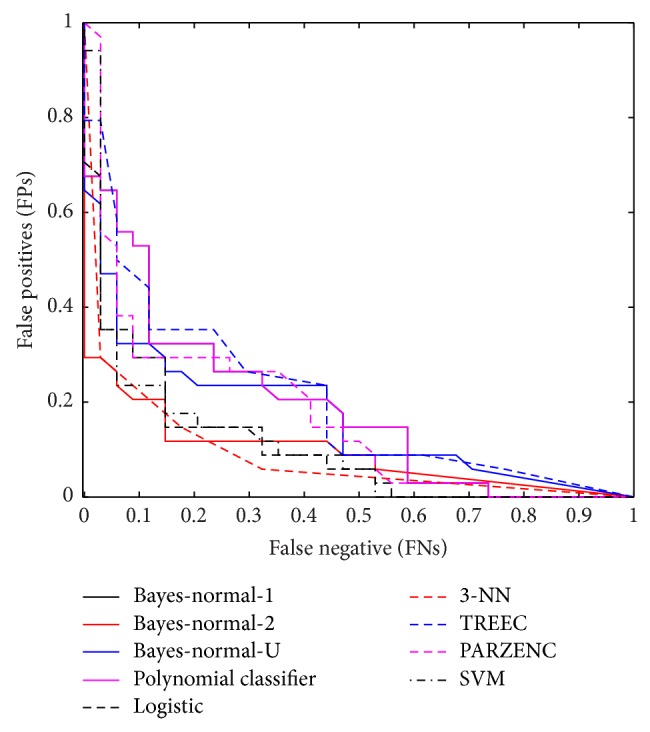
Received operator curve for top 20 uncorrelated features.

The receiver operator characteristic (ROC) curve shows the cut-off values for the false-negative and false-positive rates.  Figure 2 indicates that several of the classifiers performed reasonably well. The AUC values in Table 3 support these findings with good accuracy values for the LOGLC and KNNC classifiers.

### 6.2. Results Using Top Five Uncorrelated Features Ranked Using LDA Backward Search Feature Selection from Five Head Regions

In the second evaluation, the top five uncorrelated features extracted from five main regions across the head were used to determine whether the detection of seizures could be improved. Again, the performance for each classifier was evaluated using the sensitivity, specificity, mean error, standard deviation, and AUC values with 100 simulations and randomly selected training and testing sets for each simulation.

#### 6.2.1. Classifier Performance

As shown in Table 8, the sensitivities (seizure) for most of the algorithms improved, including the specificities values. The AUC results also showed improvements for several of the classifiers, with 93% achieved by the KNNC classifier. This is encouraging given that sensitivities are more important in this research than specificities. From the previous results, we found a 4% increase in sensitivities, a 3% increase in specificities, and a 2% increase in the performance of the KNNC classifier with other classifiers improving with similar increases.

Again, the results in Table 9 show that the global mean error has decreased by 3% using the holdout technique. The* k*-fold technique was able to decrease the global error by 6% compared with the previous evaluation, suggesting that using a region-by-region approach improves discrimination between seizureand nonseizureevents.

Overall, the mean errors produced, using all of the validation techniques, are significantly lower than the expected error, which is 171/342, that is, 50%.

#### 6.2.2. Model Selection

**Figure 3:**
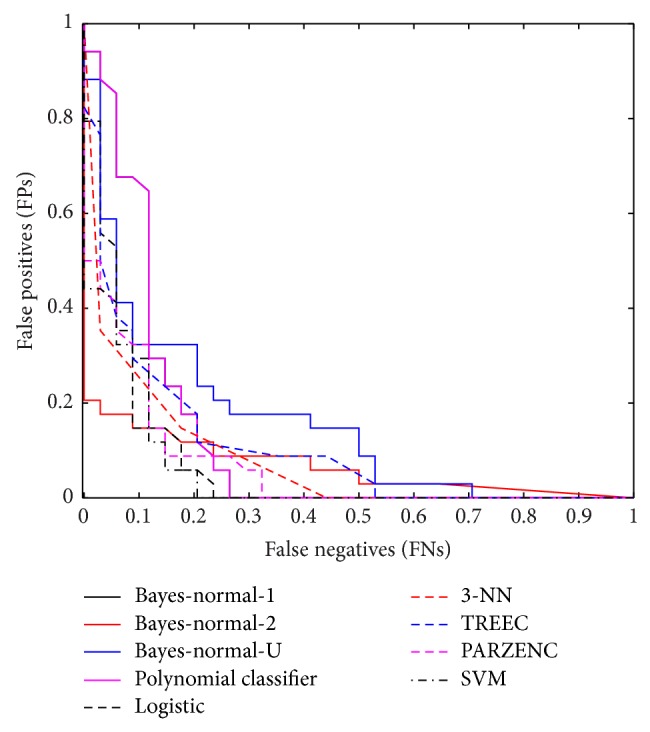
Received operator curve for top five uncorrelated features from five head regions.

Again, the ROC curve shows the cut-off values for the false-negative and false-positive rates.  Figure 3 indicates that the performance of several classifiers improved. The AUC values in Table 8 support these findings with the KNNC classifier showing a 2% increase in performance.

### 6.3. Results Using Top Twenty Uncorrelated Features Ranked Using LDA Backward Search Feature Selection and Oversampled Using SMOTE

To test whether a larger number of observations can improve on the previous set of results, the 171 seizure and 171 nonseizure records were oversampled using the SMOTE technique. The SMOTE algorithm generates synthetic samples to increase the overall size of the dataset (in this case, it doubles the number of seizureand nonseizure records). As with the first evaluation, the top 20 uncorrelated features were used with oversampling to determine whether the overall detection rate could be improved.

#### 6.3.1. Classifier Performance

Table 10 indicates that the sensitivities and specificities, for some of the algorithms, improved. Furthermore, the AUC results showed improvements with the KNNC classifier achieving 96%. The results also show that the AUC values, for several other algorithms, increased. From the previous set of results (region-by-region), we found a 2% increase in sensitivities, 3% increase in specificities, and 3% increase in the performance of the KNNC classifier.

The results in Table 11 show that the global mean error has not improved on the previous evaluation. However, the* k*-fold technique was able to decrease the global error by 4% compared with the previous evaluation, indicating that using a larger number of observations improves the discrimination between seizureand nonseizurerecords.

The results show that, using the 80% holdout method, several classifiers produced better results. Overall, the global mean errors were significantly lower than the expected error, which is 342/684, that is, 50%.

#### 6.3.2. Model Selection

**Figure 4:**
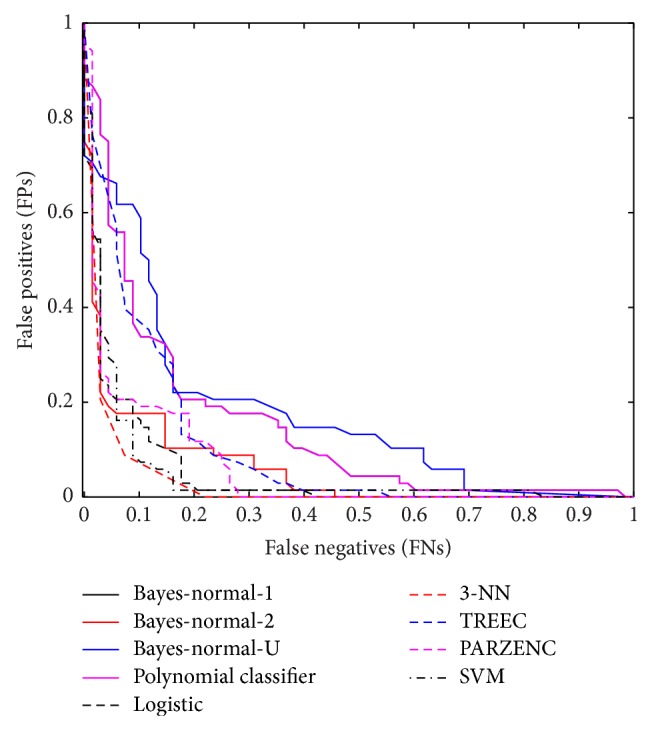
Received operator curve for top 20 uncorrelated features using SMOTE.

The ROC curve again shows the cut-off values for the false-negative and false-positive rates.  Figure 4 shows an improvement on the previous set of results.

### 6.4. Results Using Top Five Uncorrelated Features Ranked Using LDA Backward Search Feature Selection from Five Regions and Oversampled Using SMOTE

In the final evaluation, the top five uncorrelated features extracted from five main regions across the head were used with oversampling to determine whether the overall detection rate could be improved.

#### 6.4.1. Classifier Performance

Table 12 indicates that the sensitivities and specificities, for the algorithms, improved. In addition, the AUC results showed a 2% increase on the previous evaluation with the KNNC achieving 98% accuracy. The results show that the AUC values, for several other classifiers, increased. From the previous set of results, we found a 3% increase in sensitivities, 3% increase in specificities, and a 2% increase in performance for the KNNC classifier.

The results in Table 13 show that the global mean error has decreased by 3% using the holdout technique. The* k*-fold technique was able to decrease the global error by a further 3% compared with the previous evaluation. This indicates that using a region-by-region approach and a larger dataset is better at discriminating between seizureand nonseizureevents.

The final set of results shows that using the 80% holdout method, several classifiers produced better results. The best result was obtained in the final evaluation by the KNNC classifier with 93% for sensitivity, 94% for specificity, 98% for AUC, and 6% global error.

#### 6.4.2. Model Selection

**Figure 5:**
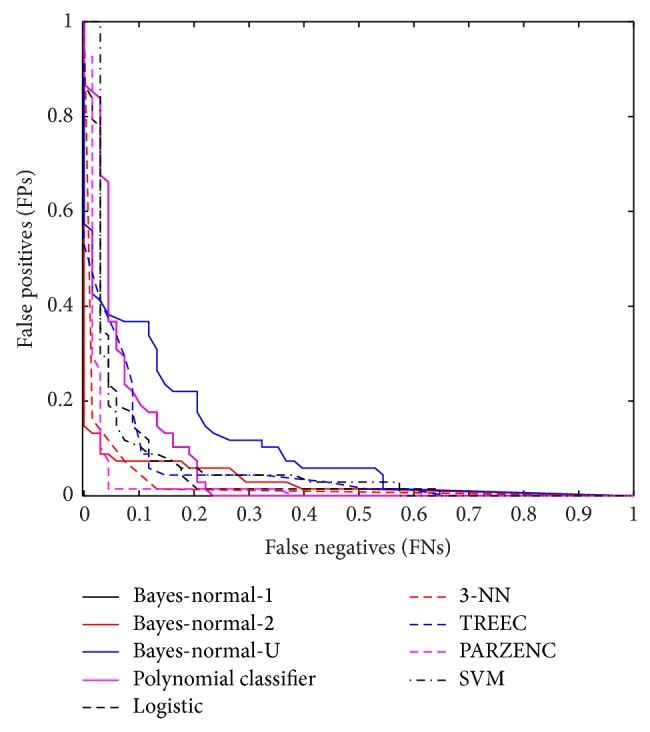
Received operator curve for top five uncorrelated features ranked using LDA backward search feature selection from five regions and oversampled using SMOTE.

The ROC curve in this final evaluation is illustrated in Figure 5, and it shows a clear improvement when compared with the previous set of evaluations.

## 7. Discussion

The study has focused on discriminating between seizure and nonseizure EEG records across a group of 24 subjects, in contrast to earlier studies that have focused on seizure detection in single individuals. The classifiers were trained using all 24 patients, allowing classification to be generalised across the whole population contained in the CHB-MIT database. To achieve this, features from all the channels that capture the EEG were used. In the initial, classification results using the top 20 uncorrelated features from the whole brain were extracted from 805 possible features using the linear discriminant analysis backward search technique to rank features (this technique was adopted because it produced the biggest AUC value, 91%, during the feature-ranking phase). This approach achieved reasonably good results, using the KNNC classifier, with 84% for sensitivity, 85% for specificity, and 91% for the AUC, with a global error of 15%.

Interestingly, the features used in this initial evaluation involved channels from the eight lobes of the brain but not the channels spreading across the centre of the scalp (*F3-C3, C3-P3, FZ-CZ, CZ-PZ, F4-C4*, and* C4-P4*). This implied that rather than having generalised seizures across the whole brain, a majority of focal seizures occurred in each of the lobes. Unlike previous studies that used the BONN dataset [43], which only contains one channel, or the FRE dataset that contains six channels and identifies focal and extra focal channels, the CHB-MIT database used in this study contains 23 channels with no information on the seizure type or location.

Using the top five uncorrelated features from EEG channels specific to the five main regions of the head improved the sensitivities and specificities, while producing high AUC values. The best classification algorithm was the KNNC classifier, which achieved 88% for sensitivity, 88% for specificity, and an AUC value of 93% with a 12% global error. This was followed closely by the SVC classifier, which achieved 85% for sensitivity, 86% for specificity, and an AUC value of 90% with a 14% global error.

The SMOTE technique was used to increase the number of seizureand nonseizure records and again to determine whether the previous results could be improved. The top 20 uncorrelated features from the whole brain were used. This improved the sensitivity, specificity, and the AUC results. The best classification algorithm was the KNNC classifier, which achieved 90% for sensitivity, 91% for specificity, and an AUC value of 96% with 9% global error. We found that using the SMOTE technique and five uncorrelated features from EEG channels specific to the five main regions of the head provided further improved sensitivity, specificity, and AUC results. The best classification algorithm was again the KNNC classifier, which achieved 93% for sensitivity, 94% for specificity, and an AUC value of 98% with 6% global error.

Comparing our results with other studies, we find that Shoeb [62] produced a better sensitivity value (96%) than those reported in this study. However, their approach utilised a SVM classifier trained and tested on an individual patient and was not concerned with the generalisation of seizures across a bigger population group. Consequently, the 93% sensitivity value produced in this paper appears to be extremely good given that our classifiers were trained and tested on data from 24 different patients not just on one. In a similar  study, Nasehi and Pourghassem [64] used a neural network and reported a sensitivity value of 98%, which again is higher than the results reported in this study. However, as with the work of Shoeb, the classifiers were trained and tested on specific patients.

In comparison with other studies that adopted a similar approach to our study, our approach produced better overall results. For instance, in [63], Khan et al. report an 83.6% specificity value, while Patel et al. [74] report 94% for sensitivity, 77.9% for specificity, and 87.7% for overall accuracy. Yuan et al. [18] report 91.72% for sensitivity, 94.89% for specificity, and 94.9% for accuracy. While Aarabi et al. [11], Acharya et al. [44], Kannathal et al. [38], and Patnaik and Manyam [73] all reported similar results. The results found in this paper can be compared in more detail with the papers listed in Table 14.

Our study produced better results than similar studies reported in the literature. Where this is not the case, a patient-specific seizure detector was used and is therefore noncomparable.

This work has potential future clinical applications in the investigation of patients with suspected seizure disorders and may be useful in the assessment of patients with nonepileptic attack disorder (NEAD). Introducing automated seizure detection technologies could help increase capacity within healthcare systems such as the UK National Health Service (NHS), which currently suffers from a chronic shortage of trained clinical neurophysiologists to interpret EEGs [98]. Tele-EEG reporting has previously been suggested as a solution, but this carries increased costs and there remain concerns over data security [99]. Automated seizure detection may therefore be a viable solution, following further work aimed at further improving accuracy.

## 8. Conclusions and Future Work

Epilepsy is one of the most common neurological conditions and one of the least understood. The seizures that characterise epilepsy are frequently unannounced and affect a sufferer's quality of life, as well as increasing the risk of injury and, in some cases, death. A strong body of evidence has suggested that these epileptic seizures can be predicted by analysis of EEG recordings.

Within a supervised-learning paradigm, this paper utilises EEG signals to classify seizure and nonseizure records. Most of the previous work in this area has focused on detecting seizures using data from individual patients. In this paper, however, the focus has been to generalise seizure detection across a group of subjects from the CHB-MIT database.

A rigorous, methodical, approach to data preprocessing was undertaken, and features were extracted from the raw EEG signals using several feature-ranking techniques. In the first evaluation, the top twenty uncorrelated features, extracted from each of the frequency bands within the EEG channels, were used to train nine classifiers. AUC values as high as 91% were achieved, with sensitivity and specificity as high as 85% when using the KNNC classifier. In the second evaluation, the top five uncorrelated features were extracted from five main regions across the head and again were used to train nine classifiers. This approach improved the AUC, sensitivities, and specificities for several of the classifiers. The highest result, achieved with the KNNC classifier, was 93% for the AUC, 88% for sensitivity, and 88% for specificity. This was closely followed by the SVC classifier, where the AUC was 90%, sensitivity was 85%, and specificity was 86%.

There were concerns that the number of observations in the CHB-MIT database was small. To test whether a larger dataset containing synthetic data would yield better results, the original CHB-MIT dataset was oversampled using the SMOTE technique to double the size of both 37 classes (342 seizures and 342 nonseizures). The same evaluations were performed again using the oversampled dataset and the top 20 uncorrelated sets of features, including the top five uncorrelated features from the five main regions of the brain. This technique improved the results with an AUC of 96%, a sensitivity of 80%, and a specificity of 91% for the KNNC classifier when using the 20 uncorrelated features. However, the best results were when the top five uncorrelated features from the five main regions were used on the oversampled dataset with an AUC value of 98%, a sensitivity of 93%, a specificity of 94%, and a global error of 6%.

Future work will include the use of regression analysis, using a larger number of observations. This would help to predict the early signs of a seizure, not just when the seizure happens. Another direction of research will include the evaluation of different parameter adjustment settings. In addition, more advanced classification algorithms and techniques will be considered, including advanced artificial neural network architectures, such as higher order and spiking neural networks. The investigation and comparison of features, such as fractal dimension and cepstrum analysis, autocorrelation zero crossing and correlation dimension, have also not been performed.

More importantly, continuous long-term EEG recordings of several hours for one subject (rather than 60-second blocks) will be investigated in future work. This will include the detection of different types of seizure activity and how well classifiers can differentiate between them.

Overall, the study demonstrates that classification algorithms provide an interesting line of enquiry, when separating seizure and nonseizure records.

## Figures and Tables

**Table 1:** Seizure information for each case.

Case	Number of seizures	Gender	Age
1	7	F	11
2	3	M	11
3	7	F	14
4	4	M	22
5	5	F	7
6	10	F	1.5
7	3	F	14.5
8	5	M	3.5
9	4	F	10
10	7	M	3
11	3	F	12
12	27	F	2
13	10	F	3
14	8	F	9
15	20	M	16
16	8	F	7
17	3	F	12
18	6	F	18
19	3	F	19
20	8	F	6
21	4	F	13
22	3	F	9
23	7	F	6
24	16	Unknown	Unknown

**Table 2:** Results for Feature Selection techniques.

AUCs for Feature Selection techniques								
AUC^knn^	AUC^knn^	AUC^svn^	AUC^knn^	AUC^treec^	AUC^knn^	AUC^loglc^	AUC^knn^	AUC^SVN^
P	q	PC1	PC2	PC1 & 2	LDAi	LDAf	LDAb	GS
90	89	83	88	87	86	88	91	88

Sensitivities for Feature Selection techniques								
SENS^knn^	SENS^knn^	SENS^svn^	SENS^knn^	SENS^treec^	SENS^knn^	SENS^loglc^	SENS^knn^	SENS^loglc^
*p*	*q*	PC1	PC2	PC1 & 2	LDAi	LDAf	LDAb	GS

83	84	53	86	80	78	76	84	76

Specificities for Feature Selection techniques								
SPEC^knn^	SPEC^knn^	SPEC^svn^	SPEC^knn^	SPEC^treec^	SPEC^knn^	SPEC^loglc^	SPEC^knn^	SPEC^loglc^
*p*	*q*	PC1	PC2	PC1 & 2	LDAi	LDAf	LDAb	GS

83	82	90	81	79	80	85	85	86

**Table 3:** List of channels for the five scalp regions.

Region	Channels
1	FP1-F7, F7-T7, FP1-F3, F3-C3, T7-FT9
2	FP2-F4, F4-C4, FP2-F8, F8-T8, T8-FT10
3	T7-P7, P7-O7, C3-P3, P3-O1
4	C4-P4, P4-O2, T8-P8, P8-O2
5	FZ-CZ, CZ-PZ, FT9-FT10

**Table 4:** Top five features for the five scalp regions.

Feature set	Description	Features
1	Top 5 features from region 1	RMS CH2 0.5–30 Hz Sample entropy CH2 0.5–4 Hz RMS CH2 4–8 Hz RMS CH2 0.5–4 Hz Sample entropy CH1 0.5–4 Hz

2	Top 5 features from region 2	RMS CH16 0.5–30 Hz RMS CH16 0.5–4 Hz RMS CH12 12–30 Hz RMS CH16 12–30 Hz RMS CH16 4–8 Hz

3	Top 5 features from region 3	RMS CH3 0.5–30 Hz RMS CH3 0.5–4 Hz RMS CH4 4–8 Hz Med Freq CH3 0.5–4 Hz RMS CH4 0.5–30 Hz

4	Top 5 features from region 4	RMS CH18 4–8 Hz RMS CH18 0.5–30 Hz RMS CH17 0.5–30 Hz RMS CH17 0.5–4 Hz RMS CH18 0.5–4 Hz

5	Top 5 features from region 5	RMS CH21 0.5–30 Hz RMS CH21 4–8 Hz RMS CH21 12–30 Hz RMS CH21 8–12 Hz RMS CH21 0.5–4 Hz

**Table 5:** Summary of classifiers considered in this study.

Classifiers	Features	Validation	Sample sizes
Density-based	Variance	Holdout cross-validation	Original (171 seizures/171 nonseizures)
Linear discriminant classifier (LDC)			
Quadratic discriminant classifier (QDC)	Root mean squares	*k*-fold cross-validation	
Uncorrelated normal density classifier (UDC)			
Linear and polynomial-based	Skewness	Sensitivity/specificity	
Polynomial classifier (POLYC)	Kurtosis		
Logistic classifier (LOGLC)	Peak frequency		SMOTE (342 seizures/342 nonseizures)
Nonlinear-based	Median frequency	Receiver operator curve	
*K*-class nearest neighbour classifier (KNNC)			
Decision tree classifier (TREEC)			
Parzen classifier (PARZENC)	Sample entropy	Area under the curve	
Support vector classifier (SVC)			

**Table 6:** Classifier performance results for top 20 uncorrelated features.

Classifier	Sensitivity	Specificity	AUC
LDC	70%	83%	54%
QDC	65%	92%	62%
UDC	39%	95%	65%
POLYC	70%	83%	83%
LOGLC	79%	86%	89%
KNNC	**84%**	**85%**	**91%**
TREEC	78%	80%	86%
PARZENC	61%	86%	54%
SVC	79%	86%	88%

**Table 7:** Cross-validation results for top 20 uncorrelated features.

Classifiers	80% holdout: 100 repetitions		Cross-validation, 5-fold, 1 repetition	Cross-validation, 5-fold, 100 repetitions	
	Mean error	SD	Mean error	Mean error	SD
LDC	0.2386	0.0506	0.2427	0.2398	0.0107
QDC	0.2179	0.0434	0.2164	0.2171	0.0064
UDC	0.3299	0.0431	0.3304	0.3310	0.0035
POLYC	0.2388	0.0507	0.2544	0.2385	0.0107
LOGLC	0.1771	0.0489	0.1813	0.1734	0.0085
KNNC	**0.1527**	**0.0401**	0.1696	0.1674	0.0148
TREEC	0.2071	0.0510	0.1959	0.2003	0.0157
PARZENC	0.2651	0.0493	0.2544	0.2640	0.0100
SVC	0.1752	0.0416	0.1608	0.1728	0.0072

**Table 8:** Classifier performance results from top five uncorrelated features from five head regions.

Classifier	Sensitivity	Specificity	AUC
LDC	78%	88%	55%
QDC	84%	86%	60%
UDC	51%	91%	70%
POLYC	78%	88%	89%
LOGLC	82%	84%	90%
KNNC	**88%**	**88%**	**93%**
TREEC	82%	81%	89%
PARZENC	81%	93%	61%
SVC	85%	86%	90%

**Table 9:** Cross-validation results from top five uncorrelated features from five regions.

Classifiers	80% holdout: 100 repetitions		Cross-validation, 5-fold, 1 repetition	Cross-validation, 5-fold, 100 repetitions	
	Mean error	SD	Mean error	Mean error	SD
LDC	0.1690	0.0419	0.1696	0.1675	0.0120
QDC	0.1493	0.0449	0.1462	0.1509	0.0088
UDC	0.2926	0.0440	0.2836	0.2940	0.0037
POLYC	0.1690	0.0419	0.1871	0.1709	0.0091
LOGLC	0.1734	0.0413	0.1696	0.1648	0.0120
KNNC	**0.1203**	**0.0339**	0.0936	0.1135	0.0101
TREEC	0.1835	0.0460	0.1988	0.1784	0.0202
PARZENC	0.1328	0.0433	0.1316	0.1325	0.0146
SVC	0.1460	0.0378	0.1316	0.1411	0.0101

**Table 10:** Classifier performance results for top 20 uncorrelated features using SMOTE.

Classifier	Sensitivity	Specificity	AUC
LDC	72%	84%	54%
QDC	64%	94%	64%
UDC	38%	95%	66%
POLYC	72%	84%	85%
LOGLC	82%	88%	92%
KNNC	**90%**	**91%**	**96%**
TREEC	87%	88%	92%
PARZENC	75%	92%	57%
SVC	82%	89%	91%

**Table 11:** Cross-validation results for top 20 uncorrelated features using SMOTE.

Classifiers	80% holdout: 100 repetitions		Cross-validation, 5-fold, 1 repetition	Cross-validation, 5-fold, 100 repetitions	
	Mean error	SD	Mean error	Mean error	SD
LDC	0.2174	0.0328	0.2237	0.2158	0.0073
QDC	0.2062	0.0286	0.2003	0.2037	0.0055
UDC	0.3322	0.0297	0.3333	0.3314	0.0020
POLYC	0.2174	0.0328	0.2266	0.2148	0.0056
LOGLC	0.1498	0.0285	0.1477	0.1469	0.0048
KNNC	**0.0959**	**0.0232**	0.0599	0.0614	0.0074
TREEC	0.1234	0.0295	0.1360	0.1227	0.0115
PARZENC	0.1620	0.0420	0.1462	0.1589	0.0130
SVC	0.1428	0.0292	0.1418	0.1412	0.0055

**Table 12:** Classifier performance results for top five uncorrelated features ranked using LDA backward search feature selection from five regions and oversampled using SMOTE.

Classifier	Sensitivity	Specificity	AUC
LDC	82%	90%	56%
QDC	87%	92%	63%
UDC	52%	91%	70%
POLYC	82%	90%	92%
LOGLC	88%	87%	94%
KNNC	**93%**	**94%**	**98%**
TREEC	90%	90%	94%
PARZENC	96%	98%	82%
SVC	90%	89%	93%

**Table 13:** Cross-validation results for top five uncorrelated features ranked using LDA backward search feature selection from five regions and oversampled using SMOTE.

Classifiers	80% holdout: 100 repetitions		Cross-validation, 5-fold, 1 repetition	Cross-validation, 5-fold, 100 repetitions	
	Mean error	SD	Mean error	Mean error	SD
LDC	0.1359	0.0291	0.1374	0.1308	0.0044
QDC	0.1060	0.0267	0.1023	0.1082	0.0043
UDC	0.2835	0.0304	0.2851	0.2881	0.0025
POLYC	0.1359	0.0291	0.1301	0.1337	0.0049
LOGLC	0.1260	0.0262	0.1213	0.1182	0.0072
KNNC	**0.0661**	**0.0198**	0.0278	0.0311	0.0049
TREEC	0.0974	0.0319	0.1082	0.0969	0.0117
PARZENC	0.0321	0.0170	0.0336	0.0341	0.0054
SVC	0.1072	0.0255	0.1067	0.1034	0.0063

**Table 14:** Seizure detection studies and classification results.

Author	Year	Dataset	Classifier	Patients	Sensitivity (%)	Specificity (%)	Accuracy (%)	FPR/h
Aarabi et al. [11]	2006	AMI	BPNN	6	91.00	95.00	93.00	1.17
Acharya et al. [68]	2012	BONN	PNN, SVM, C4.5, BC, FSC, KNN, GMM	10	94.4–99.4	91.1–100	88.1–95.9	—
Bao et al. [87]	2008	BONN	PNN	10	—	—	71–96.8	—
Chandaka et al. [88]	2009	BONN	SVM	10	92.00	100	95.96	—
Kannathal et al. [38]	2005	BONN	ANFIS	10	91.49	93.02	92.2	—
Kumar et al. [42]	2010	BONN	EN, RBNN	10	—	—	94.5	—
Kumari and Jose [89]	2011	BONN	SVM	5	100.00	100	100	0
Acharya et al. [44]	2012	BONN	SVM	10	94.38	93.23	80.9–86.1	—
Polat and Güneş [90]	2007	BONN	DTC	10	99.40	99.31	98.72	—
Polat and Güneş [69]	2008	BONN	C4.5	10	99.49	99.12	99.32	—
Song and Liò [91]	2010	BONN	BPNN, ELM	10	97.26	98.77	95.67	—
Srinivasan et al. [43]	2007	BONN	PNN, EN		—	—	100	
Subasi [92]	2007	BONN	MPNN, ME	10	95.00	94	94.5	—
Subasi and Gursoy [93]	2010	BONN	SVM		99-100	98.5–100	98.75–100	—
Übeyli [66]	2008	BONN	SVM	10	99.25	100	99.3	—
Übeyli [67]	2009	BONN	PNN, SVM, MPNN, CNN, ME, MME, RNN	10	99.20	99.78	99.2	—
Yuan et al. [46]	2011	BONN	SVM, BPNN, ELM	10	92.50	96	96	—
Zheng et al. [94]	2012	BXH	SVM	7	44.23	—	—	1.6–10.9
Khan et al. [63]	2012	CHBMIT	LDA	5	83.60	100	91.8	
Nasehi and Pourghassem [64]	2013	CHBMIT	IPSONN	23	98.00	—	—	0.125
Shoeb [62]	2009	CHBMIT	SVM	24	96.00	—	—	0.08
Acir and Güzeliş [75]	2004	DEU	SVM	7	90.30	—	—	
Rasekhi et al. [76]	2013	EUR	SVM	10	73.90	—	—	0.15
Park et al. [72]	2011	FRE	SVM	18	92.5–97.5	—	—	0.2–0.29
Patel et al. [74]	2009	FRE	SVM, LDA, QDA, MDA	21	90.9–94.2	59.5–77.9	76.5–87.7	—
Patnaik and Manyam [73]	2008	FRE	BPNN	21	91.29	99.19	—	—
Williamson et al. [71]	2011	FRE	SVM	21	90.80	—	—	0.094
Yuan et al. [18]	2012	FRE	ELM	21	93.85	94.89	94.9	0.35
Bao et al. [87]	2008	JPH	PNN	12	—	—	94.07	—
Saab and Gotman [10]	2005	MON	BC		76.00	—	—	0.34
Grewal and Gotman [95]	2005	MON2	BC	16	89.40	—	—	0.22
D'Alessandro et al. [96]	2005	PEN & BON	PNN	2	100.00	—	—	1.1
Sorensen et al. [97]	2010	RIG	SVM	6	77.8–100	—	—	0.16–5.31
Acharya et al. [68]	2012	SGR & BONN	PNN, SVM	21 + 10	—	—	99.9	—
Buhimschi et al. [22]	1998	Unknown	PNN	4	62.50	90.47	—	0.2775
Subasi [17]	2006	Unknown	DFNN	5	93.10	92.8	93.1	—
